# *Coxiella burnetii *Nine Mile II proteins modulate gene expression of monocytic host cells during infection

**DOI:** 10.1186/1471-2180-10-244

**Published:** 2010-09-20

**Authors:** Saugata Mahapatra, Patricia Ayoubi, Edward I Shaw

**Affiliations:** 1 Department of Microbiology and Molecular Genetics, Oklahoma State University, 307 Life Sciences East, Stillwater, OK, 74078, USA; 2 Department of Biochemistry and Molecular Biology, Oklahoma State University, 246C Noble Research Center, Stillwater, OK, 74078, USA

## Abstract

**Background:**

*Coxiella burnetii *is an intracellular bacterial pathogen that causes acute and chronic disease in humans. Bacterial replication occurs within enlarged parasitophorous vacuoles (PV) of eukaryotic cells, the biogenesis and maintenance of which is dependent on *C. burnetii *protein synthesis. These observations suggest that *C. burnetii *actively subverts host cell processes, however little is known about the cellular biology mechanisms manipulated by the pathogen during infection. Here, we examined host cell gene expression changes specifically induced by *C. burnetii *proteins during infection.

**Results:**

We have identified 36 host cell genes that are specifically regulated when *de novo C. burnetii *protein synthesis occurs during infection using comparative microarray analysis. Two parallel sets of infected and uninfected THP-1 cells were grown for 48 h followed by the addition of chloramphenicol (CAM) to 10 μg/ml in one set. Total RNA was harvested at 72 hpi from all conditions, and microarrays performed using Phalanx Human OneArray™ slides. A total of 784 (mock treated) and 901 (CAM treated) THP-1 genes were up or down regulated ≥2 fold in the *C. burnetii *infected vs. uninfected cell sets, respectively. Comparisons between the complementary data sets (using >0 fold), eliminated the common gene expression changes. A stringent comparison (≥2 fold) between the separate microarrays revealed 36 host cell genes modulated by *C. burnetii *protein synthesis. Ontological analysis of these genes identified the innate immune response, cell death and proliferation, vesicle trafficking and development, lipid homeostasis, and cytoskeletal organization as predominant cellular functions modulated by *C. burnetii *protein synthesis.

**Conclusions:**

Collectively, these data indicate that *C. burnetii *proteins actively regulate the expression of specific host cell genes and pathways. This is in addition to host cell genes that respond to the presence of the pathogen whether or not it is actively synthesizing proteins. These findings indicate that *C. burnetii *modulates the host cell gene expression to avoid the immune response, preserve the host cell from death, and direct the development and maintenance of a replicative PV by controlling vesicle formation and trafficking within the host cell during infection.

## Background

*Coxiella burnetii *is a Gram-negative, pleomorphic, intracellular bacterial pathogen with a worldwide distribution [1,2]. Virulent strains cause human Q-fever, which is usually marked by an acute self-limiting flu-like illness. Persistent infections usually progress into chronic disease [1,3,4]. Human infection occurs via inhalation of aerosols contaminated with *C. burnetii. *The small cell variant (SCV) form of the bacterium, which are metabolically inactive and environmentally stable, are believed to be responsible for most environmentally acquired infections. SCVs passively ingested by mononuclear phagocytes are trafficked along the endocytic pathway and associate with a variety of endocytic and autophagic markers before ultimately residing within a parasitophorous vacoule (PV) with characteristics of a secondary lysosome [1-3]. Here, they undergo a replicative lag phase of 1-2 days while differentiating into the metabolically active large cell variants (LCVs). Although they are not environmentally stable, LCVs are infectious in laboratory settings and pose a risk of causing disease. After differentiation, LCVs then undergo exponential replication for ~4 days (log phase) before beginning an asynchronous conversion back to SCVs at ~6 days post infection (PI) [5,6]. LCV replication is accompanied by a remarkable expansion of the PV, which eventually occupies the majority of the host cell [2,7].

Intracellular bacterial pathogens are known to operate by targeting and subverting vital intracellular pathways of the host [8,9]. Bacterial proteins are a key factor in this subversion of host cell molecular mechanisms [2,9-11]. Biogenesis and maintenance of the PV, interaction with the autophagic pathway, and inhibition of host cell apoptosis are all dependent on *C. burnetii *protein synthesis [2,7,12-14]. After ingestion by a host cell, *C. burnetii *PV maturation experiences a delay when compared to vacuoles carrying latex beads or dead *C. burnetii *[7,15]. This delay in phagolysosomal maturation requires ongoing bacterial protein synthesis [7]. *C. burnetii *protein synthesis is also required for the fusogenicity of *C. burnetii *containing vacuoles, PV fusion with host vesicles, and in the maintenance of a spacious PV (SPV) during logarithmic bacterial growth [7,15]. Transient interruption of bacterial protein synthesis results in cessation of SPV-specific vesicle trafficking and SPV collapse [7,15]. The *C. burnetii *PV is thought to interact with the autophagic pathway as a means to provide metabolites to the bacterium. This interaction is also a pathogen driven activity [16]. Additionally, an examination of the PV has revealed increased amounts of cholesterol in the membranes [12]. Interestingly, *C. burnetii *infected cells have been observed to dramatically increase cholesterol production. During log growth, the PV expands and is accompanied by increased transcription of host genes involved in both cholesterol uptake (e.g. LDL receptor) and biosynthesis (e.g. lanosterol synthase) [2,12].

Recently, the function of the host cell apoptotic pathway has been shown to be altered during *C. burnetii *infection. *C. burnetii *was shown to actively inhibit apoptosis in macrophages exposed to inducers of both the extrinsic and intrinsic apoptotic pathways in a bacterial protein synthesis dependant manner [14]. This antiapoptotic activity causes a marked reduction in activated caspase-3, caspase-9, and poly-ADP (ribose) polymerase (PARP) processing. Other data indicate that *C. burnetii *mediates the synthesis of host anti-apoptotic proteins A1/Bfl-1 and c-IAP2, which might directly or indirectly prevent release of cytochrome C from mitochondria, interfering with the intrinsic cell death pathway during infection [17]. Moreover, activation of the pro-survival host kinases Akt and Erk1/2 by *C. burnetii *was shown to protect infected host cells from apoptosis [18]. Despite the information on processes that appear to be affected by *C. burnetii *proteins, little is known about the host molecular mechanisms being targeted throughout the course of infection.

A common theme among bacterial pathogens, including *C. burnetii*, is the ability to secrete effector proteins into the host cell as part of their pathogenic strategy [9,10]. The possession of a type IV secretion system (T4SS) by *C. burnetii *suggests that effector proteins might be delivered to the host cell via this machinery [2,10,19,20]. As the genetic manipulation of *C. burnetii *is in its infancy, indirect approaches such as bioinformatic screens have been useful in predicting putative T4SS substrates. Recent data indicate that *C. burnetii *encodes multiple proteins with eukaryotic-like domains, including ankyrin repeat binding domains (Anks), tetratricopeptide repeats (TPRs), coiled-coil domains (CCDs), leucine-rich repeats (LRRs), GTPase domains, ubiquitination-related motifs, and multiple kinases and phosphatases [2,21,22]. Studies have shown that a number of the *C. burnetii *encoded Ank proteins are secreted into the host cell cytoplasm through the *Legionella pneumophila *T4SS [11,19,22]. Three of these proteins associate with the PV membrane, microtubules, and mitochondria, respectively, when expressed ectopically within eukaryotic cells [19].

These observations suggest that *C. burnetii *proteins directly interact and exploit mammalian intracellular pathways leading to the establishment and prolongation of the replicative niche. Here, we use the avirulent *C. burnetii *Nine Mile phase II (NMII) strain and the transient inhibition of bacterial protein synthesis as a means to elucidate host molecular mechanisms that are being actively targeted by *C. burnetii *during infection. While the *C. burnetii *NMII strain does not cause Q fever, it is a recognized model for the analysis of molecular host cell-pathogen interactions. Recent studies clearly demonstrate that the virulent Nine Mile phase I (NMI) and avirulent NMII strains grow at similar rates and are trafficked to similar intracellular vacuoles during infection of cultured monocytic cells (THP-1) as well as primary monocytes/macrophages [23,24], making NMII an excellent model for molecular studies of this unusual pathogen. In the current study, we have analyzed *C. burnetii *NMII protein induced gene expression changes in infected THP-1 cells. Using microarray technology we have examined the global transcriptional response of THP-1 cells during *C. burnetii *infection by transiently inhibiting (bacteriostatically) bacterial protein synthesis during the logarithmic phase of infection and comparing this to normal (mock treated) infections ran in parallel. Using stringent comparative microarray data analyses, we have discovered 36 previously unidentified host genes whose expression is significantly changed by *C. burnetii *proteins. Gene ontology analysis on these data was performed to define the host cell processes being targeted by this bacterium during infection.

## Methods

### *C. burnetii *and cell culture growth and infection

**Figure 1:**
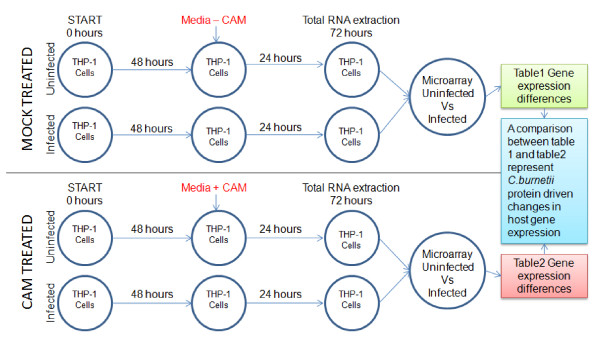
**Diagram of the experimental design for comparative *C. burnetii *infected host-cell microarrays**. The rows of the top panel are untreated and rows of the bottom panel are treated with CAM (10 μg/ml) at 48 h hpi. Total RNA harvests are performed at 72 hpi for subsequent microarray analysis.

*C. burnetii *Nine Mile phase II was grown in Vero cells (CCL-81; ATCC, Manassas, VA) and purified as previously described [20]. Non-adherent THP-1 human monocytic leukemia cells (TIB-202; ATCC) were propagated in RPMI 1640 medium (Gibco, Carlsbad, CA) supplemented with 1 mM sodium pyruvate, and 10% fetal bovine serum (FBS) at 37°C in 5% CO_2_. THP-1 cells between passages 6-10 were used in all experiments [14]. Briefly, purified *C. burnetii *NMII SCVs at a genome equivalent MOI of 15 were used to establish a synchronous infection. To ensure close host cell-bacteria contact, *C. burnetii *SCVs diluted in RPMI 1640 containing 10% FBS were incubated in 25 cm^2 ^tissue culture flasks (Becton Dickinson, Franklin Lakes, NJ) with 5 × 10^6 ^THP-1 cells in a total volume of 2.5 ml. Incubations were performed at 37°C in an atmosphere of 5% CO_2 _for 4 hours. Cells were pelleted by centrifugation at 600 g for 5 minutes, washed with fresh media and pelleted again. Cell pellets were then re-suspended in 5 ml of fresh media (final concentration = 10^6 ^cells/ml) and transferred to new 25 cm^2 ^tissue culture flasks (this represents T = 0). Cells were pelleted again at 48 hours post infection (hpi) and re-suspended in fresh media with or without the bacterial protein synthesis inhibitor chloramphenicol (CAM, a final concentration of 10 μg/ml), as needed. Cells were then incubated for an additional 24 hours for either total RNA harvest or microscopy analysis (see Figure 1). Infected and uninfected cells were handled identically and a total of three experiments (N = 3) were carried out for microarray analysis.

### Comparative microarray design and analysis

In order to perform the microarray hybridizations, two parallel infection and treatment protocols were employed. A schematic of the comparative microarray experimental design highlighting the separate treatment conditions is shown in Figure 1. Using this experimental design, a comparison was made between the THP-1 transcriptional responses of (*i*) uninfected versus *C. burnetii *NMII infected and (*ii*) uninfected versus *C. burnetii *NMII infected THP-1 cells transiently treated with bacteriostatic levels (10 μg/ml) of CAM. Briefly, infections were initiated and cultured in parallel with uninfected cells. At 48 hpi media containing CAM (10 μg/ml) was added to one set of cells (uninfected and infected THP-1 cells) and culturing was continued. The other set of cells were mock treated with normal media. Total RNA was isolated at 72 hpi from all conditions. Microarrays were performed for both conditions and the results were compared to define the host genes modulated by *de novo *synthesized *C. burnetii *NMII proteins. The 48-72 hpi time frame was used because (*i*) *C. burnetii *would be in logarithmic growth [6] and, (*ii*) previous studies have shown observable changes in PV size within *C. burnetii *infected Vero cells when treated overnight with 10 μg/ml of CAM at 48 hpi [7].

### RNA extraction, microarray hybridization and data analysis

Following the infection and treatment protocols (Figure 1), total RNA was isolated using Tri-Reagent (Ambion, Austin, TX) according to the manufacturer's recommendations. All RNA samples were DNase treated using RQ1 DNase (Promega, Madison, WI) and confirmed DNA free by PCR. RNA integrity was assessed by electropherogram using a 2100 Bioanalyzer (Agilent Technologies, Santa Clara, California). Total RNA (500 ng) from each sample was then amplified using an Epicentre^® ^Biotechnologies (Madison, WI) TargetAmp™ 1-Round AminoallylaRNA Amplification Kit, yielding approximately 6-10 μg of aminoallyl-aRNA (AA-aRNA). Alexa Fluor^® ^555-GREEN (Invitrogen, Carslbad, CA) was used to label the uninfected AA-aRNA, while Alexa Fluor^® ^647-RED (Invitrogen) was used to label the AA-aRNA from the *C. burnetii *infected cells. Labeled AA-aRNA (2 μg) with a dye incorporation efficiency range of 18-34 picomol/microgram, were mixed pair-wise and hybridized overnight to Human OneArray™ microarrays (Phalanx Biotech Group, Palo Alto, CA). Human OneArrays contain 32,050 oligonucleotides; 30968 human genome probes and 1082 experimental control probes formed as 60-mer sense-strand DNA elements. Arrays were hybridized, washed, and dried rapidly according to the manufacturer's instructions. Six hybridizations for each condition set (CAM and mock treated) were performed with three biological and two technical replicates. Signal intensity of the hybridized arrays were measured by ScanArray Express (PerkinElmer, Boston, MA, USA) and the images were processed using GenePix Pro version 4.0 (Axon, Union City, CA, USA). The processed GenePix Pro 4.0 output was further analyzed using Loess-Global intensity dependent normalization through the GenePix Auto Processor (http://darwin.biochem.okstate.edu/gpap3/) as previously described [25-27]. Normalized ratio values for each data point were averaged across the three biological replicates and two technical replicates. Significant expression differences were defined as a P-value < 0.05 and displayed as a fold change of ≥2 fold [28,29]. The microarray data were deposited at the NCBI Gene Expression Omnibus (GEO) under the platform accession number GPL6254 and the series number GSE23665. The biological function of the identified genes was determined bioinformatically by the Database for Annotation, Visualization, and Integrated Discovery (DAVID) v6.7 (http://david.abcc.ncifcrf.gov/) [30] as well as by Ingenuity pathway analysis (Ingenuity^® ^Systems, http://www.ingenuity.com). This software identifies canonical pathways within gene sets using significant associations (P < 0.05) calculated by Fisher's exact test and also by a ratio of the number of molecules from the experimental data set that maps to the pathway, divided by the total number of molecules that exists in that canonical pathway.

### Immunofluorescence microscopy

Non-adherent THP-1 cells (CAM and mock treated) were analyzed by indirect immunofluorescent antibody (IFA) microscopy. Briefly, 1 × 10^5 ^cells were cytocentrifuged onto poly-L-lysine coated slides for 2 minutes at 1000 rpm using a Shandon Cytospin^® ^4 Cytocentrifuge (Thermo Scientific) [31]. The cytospun THP-1 cells were air dried and immediately fixed using ice cold acetone for 30 seconds. The fixed preparations were then washed with PBS and stained with a rabbit antibody against whole killed *C. burnetii *NMII (primary antibody) followed by a goat anti-rabbit IgG Alexa Fluor-488 (Molecular Probes, Eugene, OR) secondary antibody. Host and bacterial DNA were also stained using 4',6-diamidino-2-phenylindole (DAPI). Microscopy was conducted using a Nikon Eclipse TE 2000-S microscope with a Nikon DS FI1 camera and NIS-ELEMENTS F 3.00 software. IMAGEJ version 1.42n (Wayne Rasband, NIH) was also used for image processing [20].

### RT-qPCR analysis

RT-qPCR was performed using gene-specific primers (shown in Additional file 1-Table S1.I), and the SYBR Green Master Mix Kit (Applied Biosystems) on an *Eppendorf Mastercycler*^® ^ep realplex (Eppendorf, Hamberg, Germany) following the manufacturer's recommendations. Briefly, first strand cDNA was synthesized using random hexamers, 1 μg of total RNA, and the SuperScript III First-Strand Synthesis System for RT-PCR (Invitrogen) as suggested by the manufacturer. Oligonucleotide primers were designed using Primer3Plus [32,33]. The primer efficiency of each primer set was determined to be within the efficiency window for the 2^-ΔΔCT ^relative fold calculation method [34]. The human β-actin gene was used as the reference gene. Paired T-Test was performed to identify statistical differences between any two conditions. Differences were considered significant at a P < 0.05.

## Results

### SPV morphology within CAM treated *C. burnetii *infected THP-1 cells

**Figure 2:**
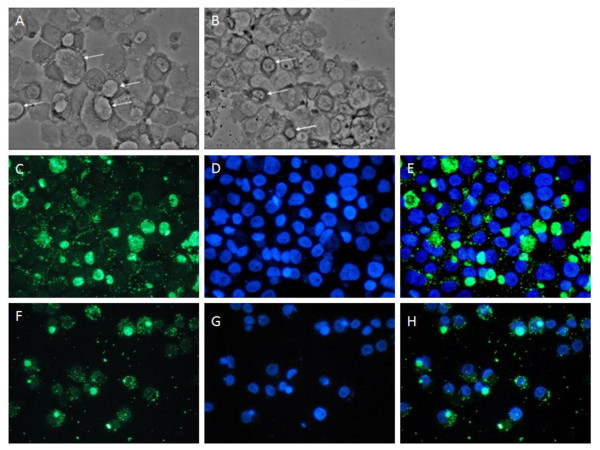
**Phase contrast and fluorescent microscopy of *C. burnetii *infected THP-1 cells**. All images are of *C. burnetii *infected THP-1 cells 72 hpi. **Top Panel**, Phase contrast microscopy. **A**, a mock treated infection. **B**, infection treated with 10 μg/ml CAM for the final 24 h. Arrows indicate PVs. **Middle Panel**, IFA microscopy images of a mock treated infection. **C**, Alexa-488 staining of *C. burnetii*. **D**, DAPI staining. **E**, merge of **C **and **D**. **Bottom Panel**, IFA microscopy images of an infection treated with 10 μg/ml CAM for the final 24 h. **F**, Alexa-488 staining of *C. burnetii*. **G**, DAPI staining. **H**, merge of **F **and **G**. 400× magnification was used for all images.

As the transient inhibition of *C. burnetii *protein synthesis within infected THP-1 cells using CAM is pivotal to testing our hypothesis, we sought to confirm that morphological changes occur to the PV of infected THP-1 cells after transient CAM treatment in a manner consistent with that observed in other cell types [35]. Using phase contrast and IFA microscopy analysis, we assessed the effect of bacteriostatic levels of CAM (10 μg/ml) on infected THP-1 cells during the log growth phase of the *C. burnetii *infectious cycle in order to coincide with subsequent microarray analysis. Robust infections (≥90% infected cells) were produced using *C. burnetii *NMII at a genome equivalent MOI of 15. Infections were either mock or CAM treated at 48 hours post infection (hpi), and then compared at 72 hpi. Figure 2 shows both phase contrast (Figure 2 top panel) and IFA microscopy (Figure 2, middle and bottom panels) images representative of the *C. burnetii *NMII infection of THP-1 cells at 72 hpi. Multiple, large SPVs can be seen in the mock treated THP-1 infections, while smaller, dense PVs are observed in the CAM treated infections. These results are in agreement with published findings where transient CAM treatment resulted in PV collapse in *C. burnetii *infected Vero cells [7]. Figure 2C-H shows a set of similarly treated infections visualized by IFA microscopy. *C. burnetii *are visualized in green (Figure 2, C and 2F) and cell nuclei are stained in blue (Figure 2, D and 2G) and the images merged (Figure 2, E and 2H). Comparing the mock and CAM treated images (Figure 2, C and 2F), a noticeable decrease in vacuole size and fluorescent intensity is observed, indicating the collapse of the SPVs within the CAM treated cells when compared to the large, SPVs observed within the mock treated cells. Comparisons of DNA samples harvested at 48 hpi (prior to CAM treatment) and 72 hpi (after 24 h CAM treatment) using qPCR determined that these samples had similar *C. burnetii *genome equivalents, indicating that the 10 μg/ml CAM concentration was acting bacteriostatically (data not shown). In addition, removal of CAM from infected cells after the 24 h transient treatment resulted in the re-establishment of large, SPVs within 48 h as observed by phase contrast microscopy (data not shown). Together, these data indicate that 10 μg/ml of CAM is able to transiently arrest *C. burnetii *protein synthesis in the THP-1 cell infection model.

### Gene expression in mock and CAM treated infected vs. uninfected THP-1 cells

As outlined in Figure 1, two whole genome RNA microarray analyses were performed resulting in the generation of two separate global gene expression profiles. A total of 784 THP-1 genes (Additional file 1- Table S1.A) were up- or down-regulated ≥2 fold in mock treated infected vs. uninfected cells while a total of 901 THP-1 Additional file 1 - Table S1.C) were up- or down-regulated ≥2 fold in CAM treated infected vs. uninfected cells. To identify the host cell functions affected by *C. burnetii *infection and proteins, these gene sets were annotated using DAVID. A modified Fisher Exact P-Value test was used to measure gene-enrichment in annotation terms. The top biological function assignments for the mapped genes (P < 0.05) expressed as the percentage of the 784 and 901 significant genes identified in the mock and CAM treated microarrays, respectively, are shown in Additional file 2- Figure S1. This figure aids in defining the prominent cell functions affected by *C. burnetii *infection and proteins. Identified as affected cell functions under both conditions are immune response, cell migration, regulation of programmed cell death, intracellular signaling cascades, regulation of cell proliferation, and cytoskeletal organization. Notable differences were observed in the percentage of genes involved with each of these functions under the mock treated and CAM treated conditions, indicating a role for *C. burnetii *proteins in changing gene expression in these pathways. Other important host cell functions influenced under the mock treated condition are protein phosphorylation, lipid storage, gas homeostasis, cell-cell signaling, and cellular ion homeostasis. While major cellular functions seen affected only in CAM treated infected THP-1 cells are cell cycle processes, cell activation, response to DNA damage, lipid (sterol and cholesterol) transport, positive regulation of cytokine biosynthetic processes, and regulation of nitric oxide biosynthetic processes. Additional file 1- Tables S1.E and S1.F list the host genes associated with each of these functions. Out of the 784 host genes identified in the mock treated data set, 62 genes were not assigned function by DAVID's biological annotation coverage. In the CAM treated infected vs. uninfected data set, 102 out of the 901 host cell genes remained unassigned.

To further define the prominent host cell pathways affected by *C. burnetii *infection and proteins, an Ingenuity pathway analysis (IPA) was performed on the 784 and 901 significant genes identified in the mock and CAM treated microarrays, respectively. IPA identifies the top canonical pathways represented in a group of genes. Additional file 1-Tables S1.G and S1.H list the top canonical pathways associated with the mRNA profiles of the mock treated and CAM treated infected vs. uninfected THP-1 cells, respectively. From the mock treated microarray set, 17 biological functions were influenced by infection while 28 functions were significantly affected by CAM treatment of infections (Additional file 1 Tables S1.E and S1.F). Many of the biological functions identified are the result of the molecular pathways identified by IPA, with several innate immune response and stress pathways implicated when *C. burnetii *protein synthesis is arrested, again indicating a role for *C. burnetii *proteins in managing the host cell response to infection.

### Comparative analysis between mRNA profiles of untreated and CAM treated uninfected/infected THP-1 cells

**Figure 3:**
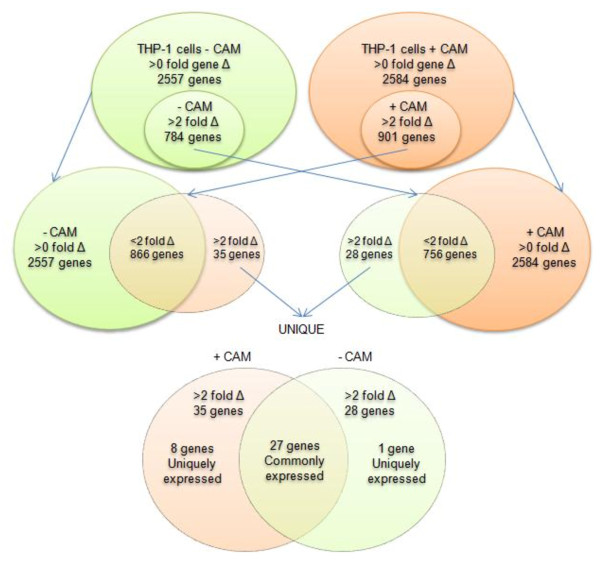
**Venn diagram of differentially expressed THP-1 genes**. A venn diagram visualization showing 784 and 901 differentially expressed host genes in *C. burnetii *infected THP-1 cells under mock (- CAM) and CAM treated (+ CAM) conditions respectively, as determined by oligonucleotide microarray analysis. Comparisons between differentially expressed genes of -CAM with the gene summary list of + CAM (>0 fold Δ = 2584 genes) and differentially expressed genes of + CAM with the gene summary list of -CAM (>0 fold Δ = 2557 genes) are also shown. The intersections (areas of overlap) indicate genes regulated in common under both conditions. Twenty-eight of the differentially expressed genes in - CAM and thirty-five of the differentially expressed genes in + CAM are modulated by *C. burnetii *protein synthesis (>2 fold difference). Of these, twenty-seven are common between the two conditions, while eight and one genes are uniquely expressed in +CAM and -CAM conditions, respectively.

In order to identify the host cell genes differentially expressed (≥2 fold) in response to *de novo C. burnetii *protein synthesis, we compared the two separate mRNA expression profiles. Microarray analysis of mock treated (-CAM), uninfected vs. infected THP-1 cells using a broad cut-off of >0 fold revealed a gene summary list of 2557 genes (P < 0.05) (Additional file 1- Table S1.B). Within this data set are the 784 genes which changed ≥2 fold (S1.A), and was considered a significant change. Using a >0 fold cut-off for the CAM treated (+CAM) uninfected vs. infected THP-1 cells, a gene summary list of 2584 genes were identified (Additional file 1 - Table S1.D). The subset of 901 genes that changed significantly (≥2 fold, S1.B) was within this large gene summary list. Figure 3 depicts a comparison of these two sets of microarray data using Venn diagrams. To eliminate the insignificantly (<2 fold) expressed genes, (*i*) the subset of significant THP-1-CAM genes (784) was cross-matched to the THP-1+CAM whole gene summary list (>0 fold) of 2584 genes and, (*ii*) the subset of significant THP-1+CAM genes (901) was cross-matched to the THP-1-CAM whole gene summary list (>0 fold) of 2557 genes. This cross comparison identified 28 genes in the THP-1-CAM subset and 35 genes in the THP-1+CAM subset that were significantly changed (≥2 fold) between the two microarray conditions. The overlapping genes from these two data sets were pooled (27 genes) and uniquely expressed genes in the -CAM (1 gene) and +CAM (8 genes) were identified. Comparing the results from these two gene subsets provided us with a list of 36 candidate host cell genes whose expression was ≥2 fold different between the mock treated (-CAM) and CAM treated (+CAM) arrays, indicating genes whose expression is modulated by *de novo *synthesized *C. burnetii *proteins.

### Host cell biological functions associated with THP-1 mRNAs modulated by *de novo C. burnetii *protein synthesis

To determine the host cell biological pathways being affected by *C. burnetii *protein synthesis, IPA was used. Analysis of the subset of thirty-six differentially expressed host genes modulated by *C. burnetii *protein(s) were classified according to the biological function they are associated with, the protein's cellular location, and its molecular function (Table 1). A majority of the proteins in this data set are predicted to reside in the cytoplasm (14 proteins) and cell nucleus (9 proteins). Six proteins are predicted to function in the extracellular space while four proteins are thought to be located on the plasma membrane. Other than cellular location, the host genes were also categorized on the basis of the expressed protein's function - i.e. enzyme, cytokine, transporter, transcriptional regulator, or other. For the thirty-six gene subset, Table 1 also lists the fold change found within the separate mock treated and CAM treated microarrays, respectively, as well as the fold difference between the arrays. *C. burnetii *infected host cells had lower RNA levels of twenty-two host genes relative to cells containing *C. burnetii *transiently inhibited with CAM. RNA levels of fourteen genes in this data set are found to be higher due to *C. burnetii *infection when compared to the CAM treated condition. Bioinformatic analysis conducted to determine possible biological functions of these *C. burnetii *modulated host genes indicates that immune response and cellular movement, cellular signaling, cellular proliferation, cell death, lipid metabolism, molecular transport, as well as vesicle trafficking, and cytoskeletal organization are affected by *C. burnetii *protein synthesis (Table 1). These data indicate that the expression of vital genes involved in cellular movement - IL8, CCL2, CXCL1, SPP1 (cytokines) are suppressed via *C. burnetii's *protein synthesis in mock treated conditions when compared to CAM treated conditions. These secretory molecules (IL8, CCL2, CXCL1, SPP1) regulate the infiltration and trafficking of immune cells. Table 1 shows other crucial host genes specifically suppressed by *C. burnetii *protein synthesis in THP-1 infection such as BCL3, CTSB and CTSL1 (apoptosis), MTSS1, SMTN and PLEKHO1 (cytoskeleton organization), APOE, PLIN2 and FABP4 (lipid metabolism), and RAB20, SOD2, PSMA8, MSC, ZFP36L1, and RORA (Miscellaneous). The prominent genes found to be up-regulated (induced) due to *C. burnetii*'s protein synthesis are ITK, DUSP9 & SKP2 (intracellular signaling), SOX11, HELLS & PGR (cell growth and proliferation) SLC22A6, CDH2, PSD4, ZNF573, CHMP5 & MRPL44 (Miscellaneous) and ANLN (cytoskeleton organization).

**Table 1:** Differentially expressed host genes modulated by *C. burnetii *protein synthesis.

Cellular Function	Gene Symbol	Cellular location	Predicted Function(s)	**-CAM**^**1**^	**+CAM**^**2**^	**FD**^**3**^
	CTSB	Cytoplasm	peptidase	3.102	6.565	↑3.463
**Apoptosis**	CTSL1	Cytoplasm	peptidase	3.173	6.914	↑3.741
	BCL3	Nucleus	transcription regulator	3.103	5.673	↑2.57

	C11ORF82	Cytoplasm	other	-1.849	-4.912	↓3.062
**Cell proliferation**	SOX11	Nucleus	transcription regulator	3.127	-2.915	↓6.042
	HELLS	Nucleus	enzyme	-1.551	-4.653	↓3.101
	PGR	Nucleus	ligand-depend. nuclear recept.	-1.539	-6.853	↓5.313

	ITK	Cytoplasm	kinase	2.752	-2.46	↓5.212
**Cell signaling**	DUSP9	Nucleus	phosphatase	-2.04	-4.388	↓2.348
	SKP2	Nucleus	other	1.581	-2.627	↓4.208

	MTSS1	Cytoplasm	other	4.389	6.986	↑2.597
**Cytoskeleton**	ANLN	Cytoplasm	other	-1.943	-4.679	↓2.735
	SMTN	Extracell. space	other	-3.319	4.006	↑7.325
	PLEKHO1	Plasma memb.	other	2.162	5.396	↑3.234

	SPP1	Extracell. space	cytokine	3.351	6.733	↑3.382
**Immune response**	CCL2	Extracell. space	cytokine	5.053	7.451	↑2.398
	CXCL1	Extracell. space	cytokine	5.221	7.275	↑2.054
	IL8	Extracell. space	cytokine	7.839	9.985	↑2.146

	FABP4	Cytoplasm	transporter	2.351	4.506	↑2.155
**Lipid metabolism**	APOE	Extracell. space	transporter	2.591	4.958	↑2.367
	PLIN2	Plasma memb.	other	3.725	5.772	↑2.047

	RAB20	Cytoplasm	enzyme	2.489	4.925	↑2.436
	FAM177B	Unknown	other	5.064	7.125	↑2.061
	SELM	Cytoplasm	other	-2.23	2.531	↑4.761
	PSMA8	Cytoplasm	peptidase	-2.494	3.212	↑5.706
	MSC	Cytoplasm	transcription regulator	3.17	5.49	↑2.32
	MRPL44	Cytoplasm	enzyme	2.775	-1.356	↓4.131
**Miscelleaneous**	CHMP5	Cytoplasm	other	1.525	-2.189	↓3.714
	RORA	Nucleus	ligand-depend. nuclear recept.	-6.756	7.147	↑13.903
	ZFP36L1	Nucleus	transcription regulator	3.815	6.842	↑3.027
	ZNF573	Nucleus	other	1.412	-3.322	↓4.734
	SLC22A6	Plasma memb.	transporter	2.097	-2.146	↓4.243
	CDH2	Plasma memb.	other	-1.626	-3.634	↓2.007
	KIAA1279	Unknown	enzyme	7.811	12.888	↑5.077
	SPATA6	Unknown	other	-2.473	19.906	↑22.379
	PSD4	Unknown	other	2.197	-2.149	↓4.346

^1^Fold change of expressed THP-1 genes in response to *C. burnetii *infection under mock treated condition.

^2^Fold change of expressed THP-1 genes in response to *C. burnetii *infection under CAM treated condition.

^3^Fold change difference increase (↑) or decrease (↓) between ^1 ^and ^2^.

### RT-q PCR analysis of THP-1 gene expression in response to mock and CAM treated *C. burnetii *infection

**Figure 4:**
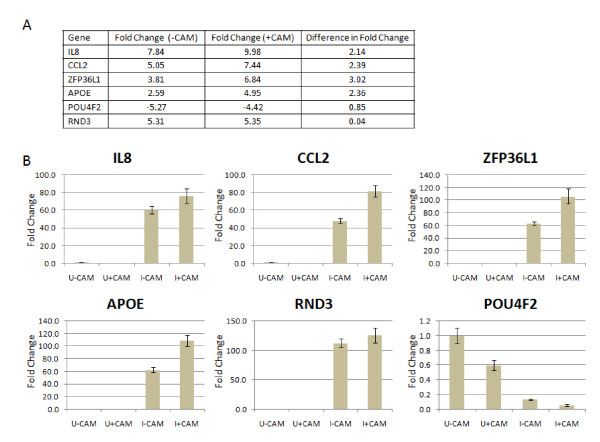
**RT-qPCR of selected genes confirms microarray expression trends**. **A**, shows the microarray data of the genes used to confirm microarray expression trends. Fold difference (-CAM) is the fold change of differentially expressed THP-1 genes in response to *C. burnetii *infection after mock treatment. Fold difference (+CAM) is the fold change of differentially expressed THP-1 genes in response to *C. burnetii *infection after CAM treatment. **B**, difference in mRNA levels in selected genes relative to β-actin. An equal amount of total RNA from each sample was analyzed by RT-qPCR. The Y-axis represents fold changes in gene expression while X axis shows the conditions under which gene expression was observed (mock and CAM treated, and uninfected and *C. burnetii *infected THP-1 cells). U-CAM, uninfected THP-1 minus CAM. U+CAM, uninfected THP-1 plus CAM. I-CAM, infected THP-1 minus CAM. I+CAM, infected THP-1 plus CAM. The results represent the mean of three biological samples and three technical replicates of each sample. Error bars represent the s.e.m.

RT-qPCR was used to validate the expression trends of selected genes identified by microarray analysis. Using the same total RNA samples utilized for the microarray hybridizations, six host genes were selected (IL8, CCL2, ZFP36L1, APOE, RND3, and POU4F2) and analyzed by RT-qPCR using the constitutively expressed β-actin gene as a comparative control. In each case, the RT-qPCR data matched the trends from the microarray analysis with respect to whether expression was increased, decreased, or unchanged. Figure 4 shows the fold expression differences of IL8, CCL2, ZFP36L1, APOE, RND3, and POU4F2 identified by microarray in mock and CAM treated experimental conditions (Figure 4A) and the subsequent RT-qPCR analysis (Figure 4B). IL8, CCL2, APOE, and ZFP36L1 represent genes that are increased in mock treated *C. burnetii *infected THP-1 cells but increase further when *C. burnetii's *protein synthesis is transiently inhibited using bacteriostatic levels of CAM. The POU4F2 gene expression is decreased similarly under both conditions and represents a THP-1 gene modulated by C. *burnetii *infection whether or not active protein synthesis is occurring. RND3 expression increases similarly in *C. burnetii *infected THP-1 cells regardless of ongoing bacterial protein synthesis. These results confirm that genes with significant mRNA expression changes by oligonucleotide microarrays analysis are differentially expressed when measured by RT-qPCR.

## Discussion

Bacterial effector proteins are crucial to the survival and growth of intracellular pathogens within the eukaryotic cellular environment. These interactions may be at a myriad of pathways or at points within a single pathway. Moreover, the growth of *C. burnetii *within the lumen of the PV would require the mediation of interactions with the host cell using effector proteins, which are predicted to be delivered by the pathogen's type IV secretion system [10,11,19]. The goal of this study was to identify host genes that are specifically manipulated by *C. burnetii *proteins. Our hypothesis was that the expression of host cell genes will be changed by infection with *C. burnetii *NMII and that the expression of a subset of these genes will be directly affected by ongoing bacterial protein synthesis. Identification of such genes will aid in the understanding of host molecular mechanisms being targeted by *C. burnetii *during growth. In order to identify the host genes regulated by *C. burnetii *proteins, we compared CAM and mock treated mRNA profiles of THP-1 cells following a 72 h infection with *C. burnetii*. Microarray data analysis shows that the majority of host genes were up- or down regulated similarly in both the mock and CAM treated array sets, suggesting that most THP-1 genes were not differentially modulated at the RNA level by active *C. burnetii *protein synthesis. We had predicted that the majority of expression changes in the host cell would be in response to the physical presence of bacteria within the cell. Using stringent analysis conditions, the transcriptional response data comparisons identified thirty-six differentially expressed genes, which were uniquely modulated by *C. burnetii *proteins. The targeting of these host genes by the pathogen indicates they may fall within pathways that *C. burnetii *needs to modulate for its own survival.

During infection *C. burnetii *replicates intracellularly, which aids in avoidance of the host immune response. Immune clearance of bacteria is largely dependent on cellular sensors called pattern recognition receptors (PRR) found on phagocytes [36]. Activated macrophages then eliminate bacteria through extrinsic or intrinsic apoptosis and/or inducing pro-inflammatory cytokines [36]. Bacteria employ indirect mechanisms to regulate cytokine production by interfering with the NFkappaB signaling pathway, which is a potent transcriptional activator of cytokines [37]. Interestingly, of the thirty-six host genes that met our criteria (Table 1) for *C. burnetii *protein driven expression changes, four are cytokines (IL8, CCL2, CXCL1 and SPP1). These secretory molecules are noted for chemo-attraction of phagocytic and lymphocytic cells [38-40]. *C. burnetii *protein(s) appear to reduce the RNA levels of each of these four genes in infected THP-1 cells relative to those found in infected cells transiently inhibited with CAM. The ability of *C. burnetii *to avoid or suppress host cytokine signaling, even transiently, may well represent an essential part of its ability to survive and cause disease by preventing communication between innate and adaptive immune cells.

Although the control and clearance of *C. burnetii *infection is T-cell dependent, specific data on T-cell activation signals are lacking [4]. One study indicated that an *in vitro *stimulation of peripheral blood mononuclear cells (PBMC) by virulent and avirulent *C. burnetii *strains cause the production of RANTES and CCL2 [41]. Using a 36 h model of *C. burnetii *infection, a DNA microarray study reported an increase in host cell expression of certain chemokines (RANTES, SCYA3, SCYA4, and IL8). The study also observed no induction of TNF-α and IL-1β after 36 h of infection, but the antimicrobial response gene encoding cytochrome b-245 (CYBB) was up-regulated [28]. In the current study, IL8 gene expression was also increased due to *C. burnetii *infection but expression was further increased when *C. burnetii *protein synthesis was inhibited, suggesting that bacterial protein(s) differentially modulate the expression of IL-8 during infection. In addition, the IL8 receptor gene (IL8RB) was found to be down regulated in mock treated, infected THP-1 cells (see Additional file 1- Table S1.A). This is the first evidence of host cell cytokine production being modulated by *C. burnetii *protein during an infection.

In addition to the immune response, *C. burnetii *has to overcome another central host defense mechanism, apoptosis. The intracellular pathogens *C. trachomatis*, *Mycobacterium tuberculosis *as well as *C. burnetii *posses mechanisms to subvert cell death pathways [13,14,42,43]. *C. burnetii *has been shown to inhibit host cell apoptosis by a mechanism that prevents cytochrome C release from the mitochondria [13]. *C. burnetii *directs the sustained activation of host pro-survival kinases Akt and Erk1/2, which are necessary for anti-apoptotic activity [13,14]. Table 1 shows that seven of the thirty-six *C. burnetii *protein modulated THP-1 genes are associated with apoptosis and cell proliferation within eukaryotic cells. *C. burnetii *protein(s) suppress the expression of three genes (BCL3, CTSB, and CTSL1), when compared to expression levels present in CAM treated THP-1 cells, which can have pro-apoptotic activities. By modulating these host genes during infection *C. burnetii *appears to promote its own survival by ensuring the survival of the host cell. The expression of the four cell proliferation/survival genes (C11ORF82, PGR, SOX11 and HELLS) are significantly reduced when *C. burnetii's *protein synthesis is inhibited during infection of THP-1 cells (Table 1). The expression of each of these genes is higher in infected cells than in infected cells where bacterial protein synthesis is inhibited, again indicating that *C. burnetii *protein(s) have an anti-cell death affect. Interestingly, our microarray analysis also shows a 4-fold expression decrease of TNFRSF10A (Death receptor 4) in mock treated infections of THP-1 cells (Additional file 1-Table S1.A). Normally, TNFRSF10A induces apoptosis by binding to TNFSF10/TRAIL ligand in cells [44], suggesting that the expression changes in *C. burnetii *infected cells may represent another means of inhibiting host cell death.

Eukaryotic host cell cytoskeleton (actin filaments, microtubules and intermediate filaments) are a common target of molecular interactions for intracellular microbial pathogens [9]. Virulent *C. burnetii *has been shown to affect F-actin reorganization in THP-1 cells [45,46]. F-actin has also been shown to be associated with PV formation and homotypic fusion of *C. burnetii *containing vacuoles, although PVs are able to acquire lysosomal markers when F-actin formation is inhibited [47]. Our analysis indicates that MTSS1, ANLN, SMTN and PLEKHO1 are differentially modulated by *C. burnetii *protein synthesis (Table 1). Compared to CAM treated THP-1 infections, the relative expression levels of MTSS1, SMTN and PLEKHO1 is lower in THP-1 mock treated infections. The relative expression of ANLN is higher in mock treated *C. burnetii *infections than in CAM treated infections. Interestingly, ANLN interacts with F-actin and is over expressed in dividing cells [48], suggesting that *C. burnetii *infection supports cell growth and division. The structure and integrity of the PV as well as host cell vesicles fusogenicity with the PV is dependent on cytoskeletol structures [47]. Finding that four out of the thirty-six genes are associated with the regulation and function of the cells cytoskeleton supports findings that the cytoskeleton is crucial to *C. burnetii *during infection.

Manipulation of cellular lipids is emerging as an important factor in infectious diseases [49,50]. Host cell cholesterol levels affect the growth of intracellular bacterial pathogens such as *Salmonellae*, *Mycobacteriae*, *Brucellae*, *Anaplasma*, and *Coxiellae *[12,50]. Little is known about cholesterol levels or imbalance in Q-fever patients, but studies at the cellular level indicate that *C. burnetii *infected Vero cells contain 73% more cholesterol than uninfected cells [12]. Table 1 lists three *C. burnetii *protein(s) modulated host genes (APOE, PLIN2, and FABP4) that are associated with lipid metabolism and regulation. These genes have lower relative expression levels in the mock treated THP-1 infections when compared to the CAM treated THP-1 infections. APOE is a multifunctional protein primarily involved in cholesterol homeostasis [51-55]. Endogenously, APOE promotes cholesterol efflux in macrophages to lower intracellular cholesterol concentrations. Macrophages deficient in APOE are severely compromised in cholesterol homeostasis [51-55]. PLIN2 and Fatty acid binding protein 4 (FABP4) are proteins that associate with lipids and fatty acids, respectively, and mediate the stabilization of lipid droplets and fatty acid transport [56,57]. An increase in cholesterol regulating proteins would be expected in response to the profound increases in the cellular concentration of cholesterol seen during *C. burnetii *infection. This makes the increase in APOE expression observed upon inhibition of *C. burnetii *protein synthesis particularly noteworthy. It seems that modulation of these key lipid homeostasis genes allows *C. burnetii *to not only suppress the loss of host cell cholesterol but to also direct lipid trafficking.

Bacterial pathogens often subvert host cell signaling pathways by introducing bacterial effector proteins that interfere with host cell phophorylation cascades [9]. *C. burnetii *dependent regulation of host cell signal transduction pathways are not well understood. Our data identified active modulation of three host cell signal transduction genes (ITK, DUSP9 and SKP2) by *C. burnetii's *protein(s). While ITK and SKP2 play significant roles in inducing host cell proliferation [58,59], DUSP9 is a mitogen-activated protein kinase phosphatase (MKP) that negatively regulates MAPK activity in mammalian cells, thus preserving the cell from apoptosis [60]. The expression of these genes are relatively higher in *C. burnetii *infected THP-1 cells compared to the expression levels found in *C. burnetii *infected THP-1 cells transiently inhibited by CAM. This suggests that *C. burnetii *protein synthesis "encourages" cell proliferation in addition to its anti-apoptotic effects as a means to preserve the host cell environment.

In addition to the outlined host cell processes, we identified a variety of genes involved in diverse functions of a host cell, which were also modulated by *C. burnetii *protein synthesis (Table 1). In this miscellaneous cellular functions category, some genes were expressed at relatively higher levels than what was expressed in CAM inhibited infected cells and are of particular interest. The PSD4 gene, which is involved in membrane recycling [61], and CHMP5, which is an essential regulator of late endosome function. CHMP5 null cells show enhanced signal transduction, protein accumulation in enlarged multi vesicular bodies (MVB) and inhibition of MVB trafficking to lysosomes [62]. In addition, we have recently found that markers of multi lamellar/multi vesicular bodies associate with membrane structures within the PV lumen during *C. burnetii *infection of Vero cells (unpublished observations). Given that *C. burnetii's *replication niche possesses markers consistent with those on late endosomes/lysosomes [2], our finding that expression of these genes are markedly lower when *C. burnetii *protein synthesis is inhibited suggests that they play a part in development and maintenance of the PV during infection. This overall manipulation of endocytosis, vesicle trafficking, and late endosome/lysosome maturation is in agreement with studies which found that inhibition of *C. burnetii *protein synthesis at any point during the life cycle changes these processes within *C. burnetii *infected cells [35,63].

## Conclusions

Through this study we have discovered thirty-six host cell genes with significant relative expression changes after transient inhibition of *C. burnetii *protein synthesis. The expression changes of these genes in the mock and CAM treatment conditions were confirmed using RT-qPCR analysis. Using bioinformatics, we have also determined the predominant host cell processes associated with these genes. Collectively, these data support our hypothesis that *C. burnetii *proteins differentially modulate host cell genes during infection. Predominant cellular functions that are modulated by *C. burnetii *proteins include (*i*) innate immune response, (*ii*) cell death and proliferation, (*iii*) vesicle trafficking and development, (*iv*) lipid homeostasis, and (*v*) cytoskeletal function. These findings indicate that *C. burnetii *actively modulates the expression of genes that may play a role in the ability of the pathogen to establish the PV, survive, and replicate within the intracellular environment.

## Competing interests

The authors declare that they have no competing interests.

## Authors' contributions

SM assisted in experimental design, carried out the experiments, participated in the microarray data analysis, and drafted the manuscript. PA assisted in experimental design of microarray assays and microarray data analysis. ES conceived the study, and participated in its design and coordination, and helped to draft the manuscript. All authors read and approved the final manuscript.

## Supplementary Material

Additional file 1 **Tables S1.A-I**. Excel file containing Tables S1.A through S1.I as individual tab-accessible tables within a single file (Supplemental Table S1.A-I). Click here for file

Additional file 2 **Figure S1. Biological function assignments of genes differentially expressed in mock and CAM treated THP-1 cells infected with *C. burnetii***. Both sets of microarray data (Additional file 1-Supplemental Tables S1.A and S1.B) containing differentially expressed genes for mock and CAM treated *C. burnetii *infections of THP-1 cells were annotated using DAVID to extract the biological functions of the listed genes. The X axis shows the percentage of differentially expressed genes associated with each annotation term while the Y axis shows the prominent biological functions (annotation terms) obtained through functional annotation of the differentially expressed genes. P-values for each annotation term are calculated using modified Fisher's exact test. A P-value cut off 0.05 or less has been used to identify biological functions. **Top panel**, shows the common host cell functions regulated under both conditions (mock and CAM treatment). **Middle panel **shows the major cellular functions affected only in *C. burnetii *infected THP-1 cells undergoing mock treatment. **Bottom panel **shows the crucial host cell functions influenced only in *C. burnetii *infected THP-1 cells undergoing CAM treatment. Click here for file
