# Supplementary figures and images for: Coxiella burnetii Nine Mile II proteins modulate gene expression of monocytic host cells during infection
Source: BMC Microbiol. 2010 Sep 20;10:244. doi: 10.1186/1471-2180-10-244 (PMC2954873; doi:10.1186/1471-2180-10-244)

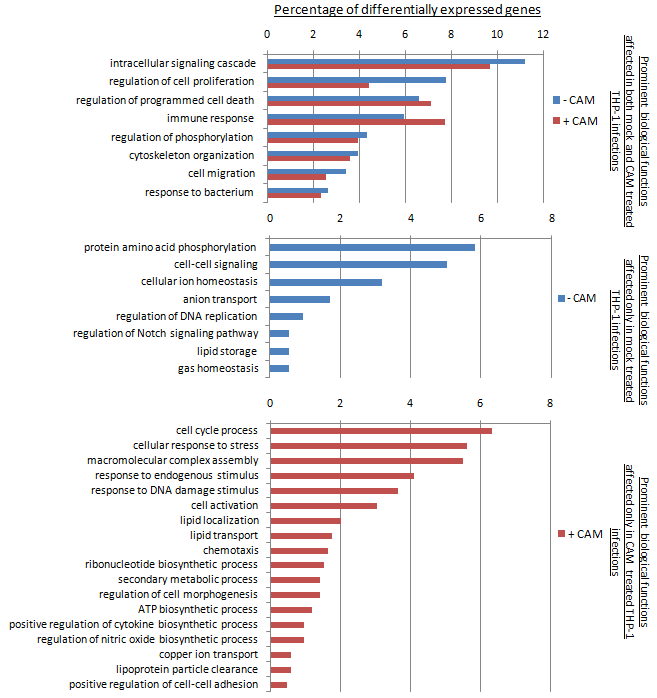

Supplement: Additional file 2 — Figure S1. Biological function assignments of genes differentially expressed in mock and CAM treated THP-1 cells infected with C. burnetii. Both sets of microarray data (Additional file 1-Supplemental Tables S1.A and S1.B) containing differentially expressed genes for mock and CAM treated C. burnetii infections of THP-1 cells were annotated using DAVID to extract the biological functions of the listed genes. The X axis shows the percentage of differentially expressed genes associated with each annotation term while the Y axis shows the prominent biological functions (annotation terms) obtained through functional annotation of the differentially expressed genes. P-values for each annotation term are calculated using modified Fisher's exact test. A P-value cut off 0.05 or less has been used to identify biological functions. Top panel, shows the common host cell functions regulated under both conditions (mock and CAM treatment). Middle panel shows the major cellular functions affected only in C. burnetii infected THP-1 cells undergoing mock treatment. Bottom panel shows the crucial host cell functions influenced only in C. burnetii infected THP-1 cells undergoing CAM treatment. [file 1471-2180-10-244-S2.DOC]
